# Targeted Delivery of Curcumin to Polyethylene-Induced Osteolysis by Magnetically Guided Zoledronate-Anchored Poly Lactic-Co-Glycolic Acid Nanoparticles via Repressing NF-κB Signaling

**DOI:** 10.3389/fphar.2020.600156

**Published:** 2020-12-04

**Authors:** Jingyi Li, Chengcheng Niu, Zichao Jiang, Zhen Zhang, Yixiao Pan, Qiqi Xing, Qi Guo, Senbo An, Yihe Hu, Long Wang

**Affiliations:** ^1^ Department of Orthopedics, Xiangya Hospital, Central South University, Changsha, China; ^2^ Department of Orthopedics, Hunan Engineering Research Center of Biomedical Metal and Ceramic Implants, Xiangya Hospital, Central South University, Changsha, China; ^3^ Department of Ultrasound Diagnosis, Second Xiangya Hospital, Central South University, Changsha, China; ^4^ Research Center of Ultrasonography, Second Xiangya Hospital, Central South University, Changsha, China

**Keywords:** polyethylene-induced osteolysis, magnetically guided, zoledronate-anchored, curcumin, targeted delivery

## Abstract

Aseptic loosening induced by periprosthetic osteolysis (PPO) is the leading complication of total joint arthroplasty (TJA) and results in patients having to receive revision surgery. However, there is still no efficient drug to prevent or even slow the pathological process. Herein, we report novel dual-targeted, curcumin-loaded Poly lactic-co-glycolic acid nanoparticles (ZSCNPs) to inhibit polyethylene-induced osteolysis. These ZSCNPs have good biocompatibility and excellent bone binding affinity. Under external magnetic field guidance, the ZSCNPs can specifically target osteolytic sites with sustained curcumin release, efficiently suppress the effect of IκB kinase, subsequently inhibit activation of the nuclear factor-kappa B (NF-κB) signaling pathway, and ultimately prevent osteoclast formation and particle-induced osteolysis. Therefore, these novel dual-targeted, drug-loaded nanoparticles could be applied as a useful strategy for targeted treatment of PPO after TJA.

## Introduction

Despite the huge clinical success of total joint arthroplasty (TJA), TJA complications, such as periprosthetic osteolysis (PPO) and infection, often lead to TJA failure and cause great pain to patients. The late stage of PPO causes aseptic loosening of prostheses, which is the most important complication after TJA, and patients often must undergo revision surgery. Some studies have shown that approximately 10–50% of revision surgeries are related to PPO (Desai and Bancroft, 2008; Beck et al., 2012). Compared with the first joint replacement, the revision surgery requires more complicated surgical techniques, longer surgical and anesthesia time, and more expensive prostheses, but the clinical effect is not satisfactory. If drug intervention can be actively carried out in the early stage of PPO, it will effectively prevent aseptic loosening of the prosthesis and greatly reduce patient pain. However, thus far, there is no effective drug for clinical treatment of PPO due to the involvement of multiple inflammatory factors in the pathological process of PPO (Termaat et al., 2005; Goodship et al., 2008).

Curcumin is a small molecular weight polyphenol that is extracted from turmeric rhizomes of the ginger family. Recent studies have shown that curcumin has a wide range of biological and pharmacological activities, such as anti-inflammatory, anti-coagulation, anti-arteriosclerosis, anti-oxidant, anti-tumor and particle-induced osteolysis activity (Anand et al., 2007; Gautam et al., 2007; Wilken et al., 2011; Chin, 2016; Kunwar and Priyadarsini, 2016; Li et al., 2017; An et al., 2018). However, curcumin has a short circulation half-life, poor hydrophilicity, and low bioavailability, which extremely limit its pharmacological activity and biomedical applications (Wilken et al., 2011). Poly lactic-co-glycolic acid (PLGA) is an FDA-approved biomaterial for controlled release formulations of injectable drugs and is used as a sustained release drug carrier that can prolong the drug half-life in blood circulation, increase drug stability and solubility, and delay the initial drug burst release (Niu et al., 2013; Tang et al., 2018; Wang et al., 2018). Therefore, loading curcumin into PLGA nanoparticles (NPs) might efficiently improve its *in vivo* bioavailability.

Zoledronate, as the latest generation of bisphosphonates, has high bone binding affinity. It can be preferentially transported to bone destruction sites, firmly adsorbs on the surface of bone trabecula, blocks the bone destruction and dissolution caused by osteoclasts (OCs), inhibits OC activity and induces OC apoptosis (Singh et al., 2015). Chaudhari et al. reported that zoledronate-anchored, drug-loaded PLGA NPs can significantly increase the bone tissue affinity of PLGA NPs and achieve targeted drug treatment of bone tissue (Ramanlal Chaudhari et al., 2012). However, due to the poor circulation of the artificial joint prosthesis in the joint cavity, intravenous drug delivery often fails to achieve an effective blood concentration at the lesion. Although intra-articular cavity drug injection can better concentrate the drug at the lesion, repeated intra-articular cavity injections greatly increase the risk of infection after THA, leading to more serious complications. To further increase the efficiency of drug accumulation in the target tissue, a passive magnetic targeting strategy can be adopted. In our previous studies, superparamagnetic iron oxide (SPIO), as a physical targeting moiety, was encapsulated in drug-loaded PLGA and showed a wonderful magnetic response under an external magnetic field (Wang et al., 2018). Therefore, it is highly desirable to design a zoledronate- and SPIO-anchored PLGA to efficiently deliver curcumin to the site of PPO under an external magnetic field.

**FIGURE 1:**
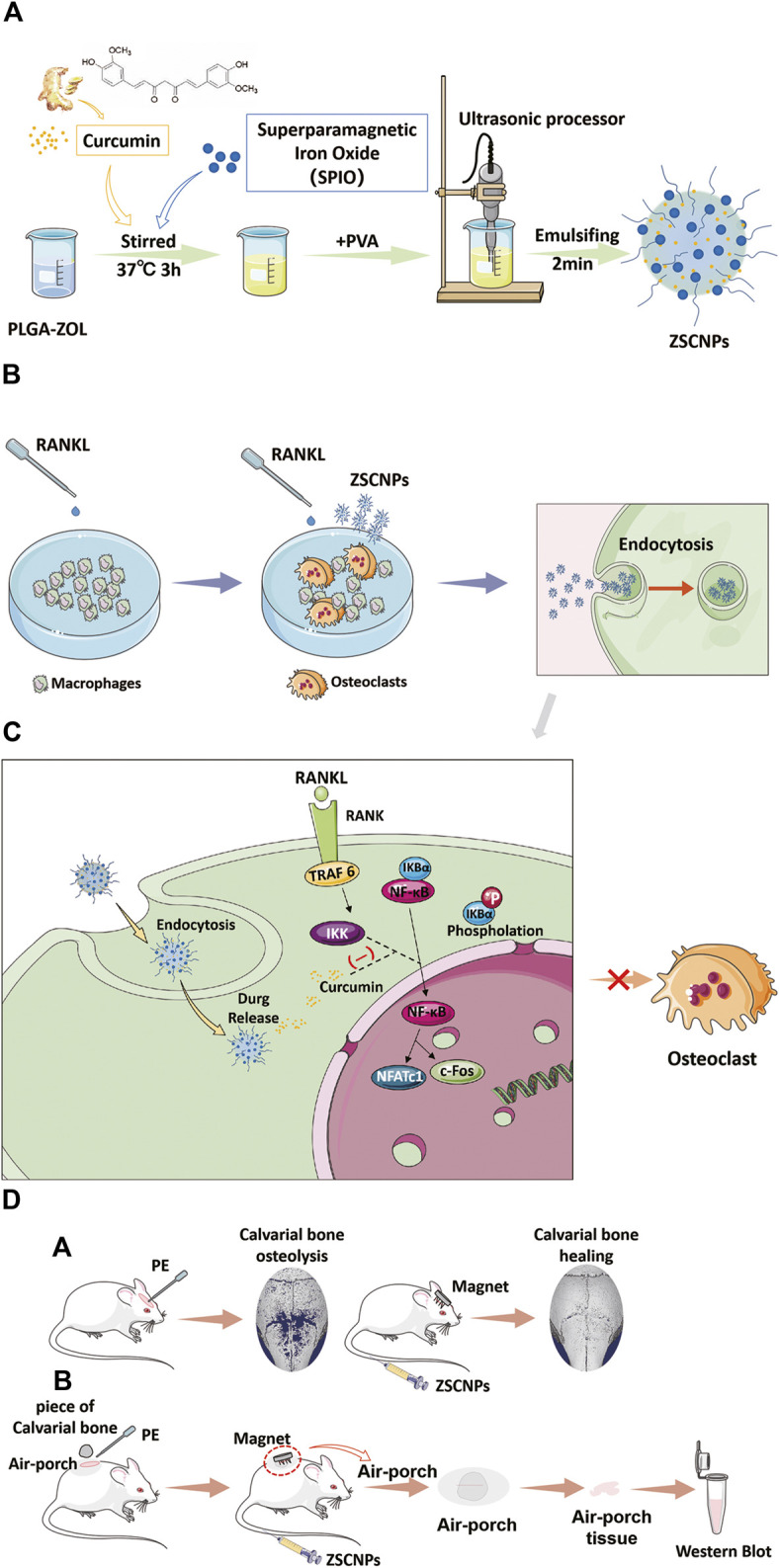
Schematic illustration of the construction of ZSCNPs and how to suppress the effect of IκB kinase for inhibiting the activation process of NF-κB signaling pathway, and preventing osteoclast formation and particle-induced osteolysis. **(A)** Preparation of ZSCNPs. **(B)** ZSCNPs inhibits receptor activator of NF-κB ligand (RANKL)-induced osteoclastogenesis in RAW 264.7 cells. **(C)** Curcumin inhibits RANKL-induced NF-κB activation. **(D)** ZSCNPs inhibits particle-induced osteolysis under an external magnet field with different models: **(a)** mouse calvarial osteolysis model; **(b)** mouse air-porch model.

On the basis of our previous research, we found that the incidence of PPO is closely related to the receptor activator of NF-κB (RANK)/receptor activator of NF-κB ligand (RANKL)/osteoprotegerin system, and the RANKL/OPG ratio in PPO is significantly increased (Wang et al., 2016). Meanwhile, NF-κB activation plays a vital role in the pathogenesis of PPO (Jiang et al., 2016), IκB kinase (IKK) plays a key role in NF-κB activation, and curcumin can inhibit IκBα phosphorylation and degradation by inhibiting the effect of IKK, finally achieving inhibition of the entire NF-κB activation process (Bharti et al., 2004). Studies have shown that inhibiting the activity of IKK can lead to repressing the phosphorylation of the p65 subunit of NF-kB, thereby inhibiting the nuclear ectopic of NF-kB to suppress downstream pathways (Fiume et al., 2015; Pontoriero et al., 2019). Furthermore, our group verified that curcumin can inhibit the entire NF-κB activation process by inhibiting the effect of IKK, preventing polyethylene (PE)-induced osteolysis and bone loss, and repressing the RANK/c-Fos/nuclear factor of activated T cells cytoplasmic 1 (NFATc1) signaling pathway (An et al., 2018). However, to the best of our knowledge, no studies have reported on the benefits of curcumin-loaded NPs in inhibiting PE-induced osteolysis. Herein, we report magnetically targeted, zoledronate-anchored, dual-targeted, drug-loaded nanoparticles (ZSCNPs) for treatment of PE-induced osteolysis under an external magnetic field and the mechanism (Figure 1). Because of the wonderful bone binding affinity, efficient sustained curcumin release in osteolytic lesions and obvious inhibition of OC formation and particle-induced osteolysis, these novel dual-targeted drug-loaded NPs have potential for targeted treatment of PPO after TJA.

## Materials and Methods

### Materials

PLGA (lactide: glycolide = 50:50, Mw = 10,000) and curcumin with a purity >98% were purchased from Sigma-Aldrich (United States). Oleic acid-treated SPIO NPs were obtained from Ocean Nanotech Co. Ltd. (United States). Zoledronate (ZOL) was obtained from Energy Chemical (China), and NH_2_-PEG-NH_2_ was purchased from Ponsure Biotechnology (China). All experiments were approved by the Ethics Committee of Xiangya Hospital, Central South University, China.

### Synthesis of Poly Lactic-Co-Glycolic Acid-Zoledronate

PLGA-ZOL was fabricated following a published study (Ramanlal Chaudhari et al., 2012). First, conjugation of PEG and PLGA was performed using N-hydroxysuccinimide (NHS) and dicyclohexylcarbodiimide (DCC) to activate PLGA, and then, the equivalent molar ratio of NH_2_-PEG-NH_2_ was added. The formed PLGA-PEG-NH_2_ was further conjugated with ZOL using N, N′-carbonyldiimidazole (CDI). ZOL (100 mg), CDI (90 mg) and triethylamine (TEA, 1 mg) were dissolved in dimethyl formamide (DMF) and tightly closed under nitrogen flow for 24 h at 60°C; the precipitates were used to activate ZOL and washed twice with acetonitrile. Then, the activated ZOL, PLGA-PEG-NH_2_ and TEA were dissolved in dimethyl sulfoxide (DMSO) in a tightly closed flask under nitrogen flow and reacted for 12 h. The mixture was freeze-dried after purification, and proton nuclear magnetic resonance (^1^HNMR) spectroscopy was used to confirm successful conjugation of PLGA-ZOL.

### Preparation of Zoledronate-Anchored, Dual-Targeted, Drug-Loaded Nanoparticles

The ZSCNPs were fabricated according to previous reports (Niu et al., 2013; Wang et al., 2018). In brief, PLGA-ZOL (100 mg), SPIO NPs (6.2 mg) and curcumin (5 mg) were added to 3 mLlof chloroform and stirred well. Then, 15 ml cold PVA solution (5% w/v) was added, and the mixture was emulsified with an ultrasonic processor and washed with phosphate-buffered saline (PBS) several times. The SCNPs were similarly prepared with PLGA instead of PLGA-ZOL, the ZCNPs were prepared without SPIO, and the other steps were the same as those for the ZSCNPs.

### Characterization of Zoledronate-Anchored, Dual-Targeted, Drug-Loaded Nanoparticles

The structure of the ZSCNPs was examined using transmission electron microscopy (TEM; H-7600, Hitachi), and NPs without SPIO loading (ZCNPs) were used as a control. The size distribution and surface charge of the ZSCNPs were analyzed using a dynamic light scattering analyzer (Malvern Nano ZS, United Kingdom). The magnetic feature of ZSCNPs was tested with a magnet. The presence of curcumin in the ZSCNPs was verified using a UV-Vis-NIR spectrophotometer (Cary 5000, Agilent, United States).

### Drug Loading and Release

To determine the drug loading capability of ZSCNPs, freeze-dried ZSCNPs (10 mg) were dissolved in 2 ml of DMSO, and the curcumin encapsulation efficiency (EE %) and loading efficiency (LE %) were measured using HPLC and calculated as follows (Eqs 1, 2):

$$\text{EE}\left(\%\right)=W_{1}/W_{2}\times 100\%$$ (1)

$$\text{LE}\left(\%\right)=W_{1}/W_{3}\times 100\%$$ (2)

where *W*_1_, *W*_2_ and *W*_3_ are the encapsulated drug, total added drug amount, and the weight of the NPs, respectively.

To verify the drug release property of ZSCNPs, freeze-dried ZSCNPs (50 mg) were reconstituted in 5 ml of Tris-HCl buffer (pH 7.4), transferred to a dialysis bag and placed in a reservoir of Tris-HCl buffer. Then, the dialysate was harvested and extracted with DMSO at different time points. The concentration of curcumin in each dialysate was measured, and the cumulative drug release was calculated using high-performance liquid chromatography (HPLC; LC-2A, Shimadzu).

### Cytotoxicity and Viability Study

The cytotoxicity of cells treated with ZSCNPs was determined using cell counting kit (CCK-8) assays. Mouse macrophage RAW264.7 cells and human dermal fibroblast (HDF) cells were seeded in 96-well plates for 12 h at 37°C. One hundred milliliters of ZSCNP suspensions at different doses (containing 0.4, 0.8, 1,6, 3.2, 8.0, and 16.0 μg/ml of curcumin) were added for 24 or 72 h of incubation, and the same volume of complete medium was added as a control for the CCK-8 study. To verify the viability of cells cultured with ZSCNPs, RAW264.7 cells were randomly divided into three groups: (1) PBS; (2) Cur NPs; (3) ZSCNPs. The concentration of Cur NPs or ZSCNPs was 500 μg/ml. Cells were stained with Hoechst 33342 and propidium iodide (PI) and imaged with an inverted fluorescence microscope.

### Dual Targeting Efficacy Study

To better evaluate the zoledronate anchoring ability and/or magnetic targeting ability of ZSCNPs, mouse macrophage RAW264.7 cells were seeded in culture dishes for 12 h. Cells were randomly divided into four groups and co-incubated with NPs: (1) SCNPs (non-targeting); (2) SCNPs + MF (magnetic targeting); (3) ZSCNPs (zoledronate anchoring); (4) ZSCNPs + MF (dual targeting). All the NPs were labeled with DiI, and the concentrations were 500 µg/ml. Round magnets (maximum MF strength of 6.6 Gs) were placed under culture dishes for 2 h for MF targeting as previously described (Wang et al., 2018). After 2 h of incubation, the cells were washed, stained with DAPI for 15 min and imaged via confocal laser scanning microscopy (CLSM; LSM 510 META, Carl Zeiss).

### Receptor Activator of NF-κB Ligand-Induced Osteoclastogenesis in RAW 264.7 Cells

Mouse macrophage RAW264.7 cells were cultured in 48-well plates and induced with or without 40 ng/ml M-CSF and 150 ng/ml RANKL for an additional 6 and 7 days. Cells treated with M-CSF and RANKL were randomly divided into three groups: (1) PBS (blank group); (2) Curcumin (24.3 μg/ml of curcumin); (3) ZSCNPs (500 µg/ml, containing 24.3 μg/ml of curcumin). Cell medium with M-CSF and RANKL was changed every day continuously until the seventh day. Cells not treated with M-CSF and RANKL were used as the blank group. On the seventh day, cells were fixed in 4% paraformaldehyde, washed with PBS, and then subjected to tartrate-resistant acid phosphatase (TRAP) staining using a leukocyte acid phosphatase cytochemistry kit (Sigma-Aldrich). TRAP-positive cells were defined as containing three or more nuclei and counted as mature OCs using a light microscope, and the TRAP activity assay was performed as previously described (An et al., 2018; Park et al., 2018; Ouyang et al., 2019).

### Animal Model

Male BALB/c mice were obtained from the Medical Experimental Animal Center of Central South University (Changsha, China), and all animal experiments were approved by the Animal Care and Use Committee of Central South University. Two animal models were constructed to evaluate the inhibitory effect of the NPs on OC formation and particle-induced osteolysis and to explore the mechanism.

In the first method, the skin and periosteum on the calvarial surface of BALB/c mice were cut; a sterilized PE suspension was directly injected into the surface of the skull; and then, the skin was sutured. After 14 days, the mouse calvarial osteolysis model was successfully established, and the calvaria were assessed via fluorescence imaging, micro computed tomography (CT) examination, *in vivo* pharmacokinetics analysis and TRAP analysis (Supplementary Figure S1).

In the second method, after hair removal on the dorsum of BALB/c mice, 2 ml of aseptic air was subcutaneously injected into the skin gap on the back with a syringe every 2 days to form an air-porch. After three air injections, the skull fragments from the other mice were trimmed to 4 × 4 mm in size and implanted into the prepared air-porch. Then, 0.5 ml of 1 mg/ml PE suspension was injected into the prepared air-porch. After 14 days, the air-porch bone graft model was successfully established, and the air-porch tissues were used for western blot analysis (Supplementary Figure S2). Meanwhile, air-porch model mice without PE injection were used as the control group.

### *In Vivo* Pharmacokinetics

Sixty calvarial osteolysis BALB/c mice were injected with 200 µl of SCNPs and ZSCNPs (10 mg/ml) through the tail vein (n = 30 in each group), and the ZSCNPs group was set with a magnet (maximum MF strength = 24.5 Gs) on the calvarial surface (Supplementary Figure S3). Submandibular venous blood was collected from the mice at different time points (0, 0.1, 0.5, 1, 2, 6, 12, 24, 48, and 72 h) for UV-Vis-NIR spectrophotometry analysis as previously described (Wang et al., 2020). The curcumin content in the blood was calculated from a standard curve.

### *Ex Vivo* Fluorescence Imaging

Nine healthy BALB/c mice and forty-five calvarial osteolysis mice were used for *ex vivo* fluorescence imaging at different times (24, 48 and 72 h). At each time point, three healthy mice were used as a normal group (1), and fifteen calvarial osteolysis mice were randomly divided into five groups (n = 3 in each group): (2) saline; (3) SCNPs; (4) SCNPs + MF; (5) ZSCNPs; (6) ZSCNPs + MF. To examine the MF effect of ZSCNPs, a magnet (maximum MF strength = 24.5 Gs) was placed on the calvarial surface for 24, 48 or 72 h in groups 4 and 6. Two hundred microliters of 10 mg/ml NPs or saline was intravenously injected into the mice. Then, the calvarial bones were collected for *ex vivo* fluorescence imaging at 24, 48 and 72 h. The average fluorescence intensities were calculated using a Bruker *In-Vivo* FX PRO fluorescence tomography system (Switzerland).

### Micro CT Imaging and Histopathological Analysis

Three healthy BALB/c mice and fifteen calvarial osteolysis mice were used in micro CT imaging. Three healthy mice were used as a normal group (1), and fifteen calvarial osteolysis mice were randomly divided into five groups (n = 3 in each group): (2) saline; (3) SCNPs; (4) SCNPs + MF; (5) ZSCNPs; (6) ZSCNPs + MF. To maximize the therapeutic effect of NPs, 200 µl of 10 mg/ml NPs or saline was intravenously injected into the mice every 48 h, and a magnet (maximum MF strength = 24.5 Gs) was placed on the calvarial surface for 48 h in groups 4 and 6. After 14 days, blood samples were collected for serum biochemistry assays. The calvarial bones were collected for micro CT imaging (Viva CT-80, SCANCO Medical AG, Zurich, Switzerland), and relevant bone volume fractions (bone volume/tissue volume, BV/TV, %) were obtained to analyze the effects of ZSCNPs on calvarial osteolysis mice and were calculated as described previously (Panmekiate et al., 2015; Huynh et al., 2017; An et al., 2018; Ouyang et al., 2019). Then, the samples were subjected to TRAP staining to assess OC formation. Simultaneously, the heart, liver, spleen, kidney and lung organs were harvested and stained with H&E.

### Western Blot Analysis

Three control air-porch mice and fifteen air-porch bone graft mice were used for western blot analysis. Three control air-porch mice without PE induction were used as the control group (1), and fifteen air-porch bone graft mice were randomly divided into five groups (n = 3 in each group): (2) saline; (3) SCNPs; (4) SCNPs + MF; (5) ZSCNPs; (6) ZSCNPs + MF. To maximize the therapeutic effect of NPs, 200 µl of 10 mg/ml NPs or saline was intravenously injected into the mice every 48 h, and a magnet (maximum MF strength = 24.5 Gs) was placed on the air-porch surface for 48 h in groups 4 and 6. After 14 days, the whole air-porch samples were collected and the proteins in air-porch wall tissues were extracted for further assays. The collected proteins were separated via sodium dodecyl sulfate-polyacrylamide gel electrophoresis, transferred to polyvinylidene fluoride membranes, and blocked with bovine serum albumin in Tris-buffered saline containing 0.05% Tween 20. Rabbit anti-c-Fos, rabbit anti-NFATc1, rabbit anti-IκBα, mouse anti-*p*-IκBα and mouse anti-β-tubulin primary antibodies were incubated with membranes at 4°C overnight, followed by incubation with fluorescent secondary antibodies for 2 h at room temperature. An enhanced chemiluminescence system (Bio-Rad Laboratories, Hercules, CA) was utilized to measure the levels of specific proteins after different treatments.

### Statistical Analysis

Continuous data are expressed as the mean ± SD and were compared via ANOVA. *p < 0.05 was considered to indicate a significant difference.

## Result and Discussion

### Synthesis and Characterization of Zoledronate-Anchored, Dual-Targeted, Drug-Loaded Nanoparticles

**FIGURE 2:**
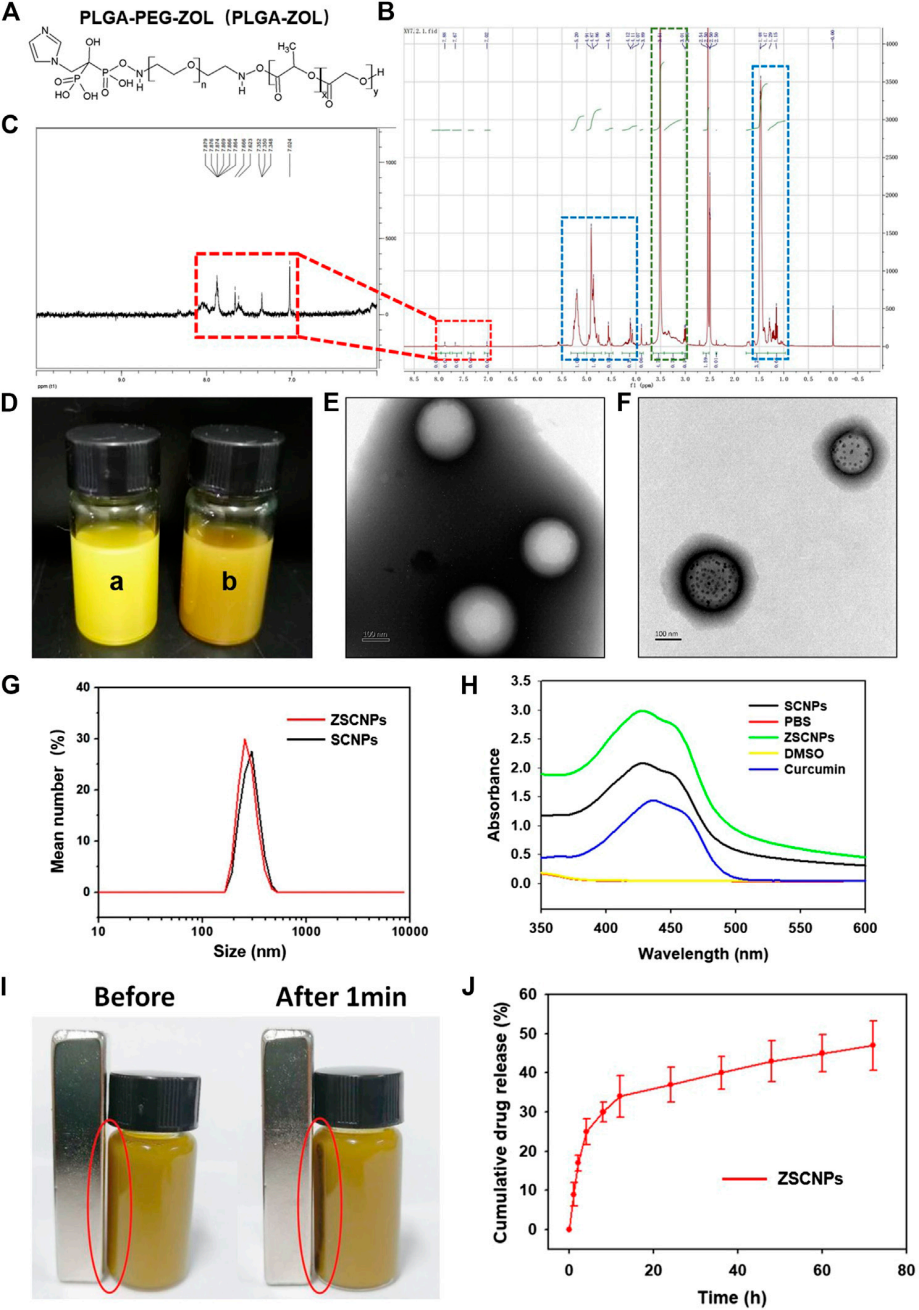
**(A)** Chemical structure and **(B,C)** 1HNMR spectra of poly lactic-co-glycolic acid-ZOL. **(D)** The photographs of ZCNPs [without superparamagnetic iron oxide (SPIO) NPs] and ZSCNPs, the ZCNPs appeared a bright yellow color **(a)**, the ZSCNPs presented a brown yellow color with the addition of SPIO nanoparticleS (NPs) **(b)**. The TEM examination of ZCNPs **(E)** and ZSCNPs **(F)**, the ZCNPs had a uniform spherical shape with an average size of 200 nm, while many black dots which respected SPIO NPs were dispersed in the shells of ZSCNPs. **(G)** The average diameters of the ZCNPs and ZSCNPs. **(H)** UV-Vis-NIR absorption spectra of ZSCNPs. **(I)** Magnetic response of ZSCNPs in an external magnetic field. **(J)** Curcumin release from ZSCNPs in 72 h.

The PLGA-PEG-ZOL (PLGA-ZOL) was synthesized through two steps following a published report (Ramanlal Chaudhari et al., 2012). The chemical formula of PLGA-ZOL is shown in Figure 2A. In the ^1^HNMR spectra of PLGA-ZOL (Figure 2B), the blue dashed circle indicates the chemical shift bonds of PLGA (1.0–1.5 and 4.5–5.5 ppm), the green dashed circle indicates the chemical shift bond of PEG (3.0–3.8 ppm), and the red dashed circle indicates the chemical shift bond of the NH-bond in PLGA-PEG-NH_2_ and the ring hydron bond in ZOL (7.0–8.0 ppm), which are magnified in Figure 2C, confirming that ZOL was successfully conjugated with PLGA-PEG-NH_2_. Photographs of the ZCNPs (without SPIO NPs) and ZSCNPs are shown in Figure 2D. The ZCNPs appeared to be a bright yellow color a), and the ZSCNPs presented a brown yellow color with the addition of SPIO NPs b). Then, the structures of ZCNPs and ZSCNPs were characterized via TEM examination (Figures 2E,F). Figure 2E shows that the ZCNPs had a uniform spherical shape with an average size of 200 nm, while many black dots representing SPIO NPs were dispersed in the shells of ZSCNPs (Figure 2F). The average diameters of the ZCNPs and ZSCNPs were 220 and 230 nm, respectively, which indicated that the SPIO addition into NPs did not affect the size of ZSCNPs (Figure 2G). Additionally, the surface zeta potentials of the ZCNPs and ZSCNPs were −5.7 and −7.3 mV, respectively, confirming that the addition of SPIO NPs had a slight negative effect on the surface zeta potential of ZSCNPs. In the UV-Vis-NIR absorption spectra, ZSCNPs, SCNPs and curcumin displayed an absorption peak at approximately 425 nm, confirming that curcumin was successfully loaded into the NPs (Figure 2H). As shown in Figure 2I, the magnetic responsiveness of the ZSCNPs was confirmed through application of an external magnetic field (MF). After 1 min, many dark NPs quickly accumulated toward the surrounding MF, showing wonderful magnetic responsiveness. Then, the drug loading capacity and the drug release characteristic of ZSCNPs were evaluated using HPLC. The EE % and LC % of curcumin were 63.66 and 4.86%, respectively. As shown in Figure 2J, the cumulative curcumin release rate of the ZSCNPs reached 34% in the first 12 h, and after 12 h, the release rate was relatively slow and reached 47% at 72 h, indicating that curcumin release from the ZSCNPs showed a sustained release effect after PLGA coating, which is consistent with previous studies (Ramanlal Chaudhari et al., 2012; Niu et al., 2013; Wang et al., 2018).

### Cell Experiments With Zoledronate-Anchored, Dual-Targeted, Drug-Loaded Nanoparticles

**FIGURE 3:**
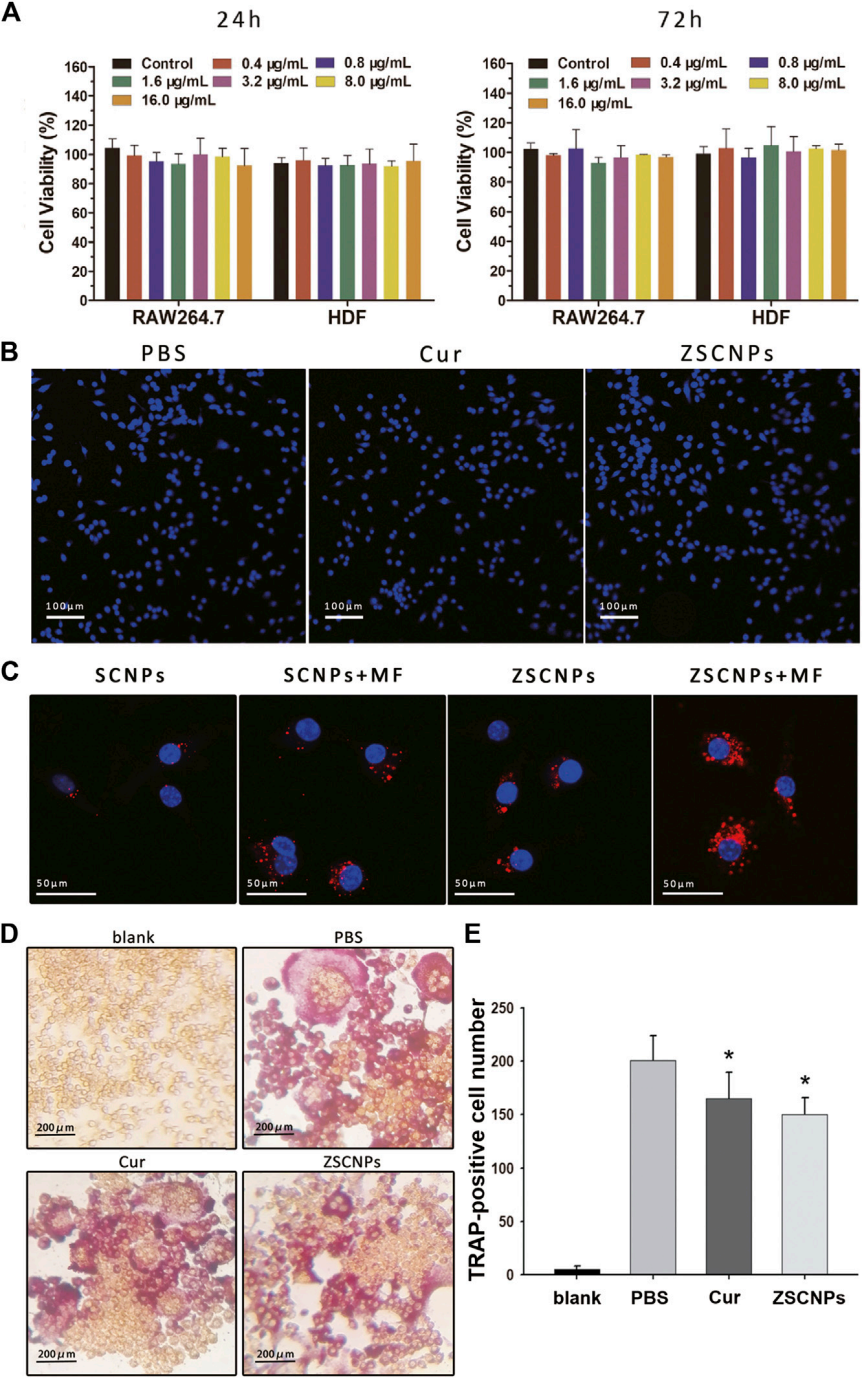
Cell experiments on ZSCNPs. **(A)** Cytotoxicity assays of RAW264.7 cells and human dermal fibroblast cells incubated with different concentrations of ZSCNPs for 24 and 48 h. **(B)** Hoechst 33342/PI co-stained of RAW264.7 cells after different treatments of PBS, curcumin and ZSCNPs (scale bar = 100 µm). **(C)** Fluorescence images of 4T1 cells incubated with ZSCNPs or SCNPs with or without magnetic targeting (MF) (scale bar = 50 µm). **(D)** *In vitro* osteoclast formation of RAW264.7 cells after receptor activator of NF-κB ligand and ZSCNPs treatment (scale bar = 200 µm). **(E)** Quantification of osteoclastogenesis. *p < 0.05 compared with PBS group.

The cytotoxicity of ZSCNPs was assessed using CCK-8 assays. There was no obvious decrease in cell viability at 24 h or 72 h with increasing concentrations of ZSCNPs (containing from 0.4 to 16.0 μg/ml curcumin) in RAW264.7 cells or HDF cells, indicating that ZSCNPs were non-cytotoxic (Figure 3A, *p* > 0.05). Subsequently, the viability of cells cultured with ZSCNPs was further verified via fluorescence imaging. The concentration of Cur NPs or ZSCNPs was 500 μg/ml (equivalent to 24.3 μg/ml of curcumin). Cells were stained with Hoechst 33,342 (blue fluorescence labeling live and dead cells) and PI (red fluorescence labeling dead cells) and imaged with an inverted fluorescence microscope. There were no obvious dead cells (red fluorescence) after co-culture with Cur NPs or ZSCNPs, demonstrating that both Cur NPs and ZSCNPs were safe for cells (Figure 3B).

An intracellular uptake study of ZSCNPs was carried out using CLSM to measure the zoledronate anchoring ability and/or magnetic targeting ability. DiI-labeled SCNPs or ZSCNPs emitted red fluorescence. As shown in Figure 3C, the red fluorescence intensity was significantly stronger in the dual-targeting group than in the zoledronate-anchoring group, magnetic targeting group and non-targeting group, reflecting that not only zoledronate anchoring but also magnetic targeting contribute to aggregation of the NPs, which benefits drug release in the target region.

To investigate the inhibitory effects of ZSCNPs on OC formation and function, TRAP staining assays of RAW264.7 cells were performed after different treatments. After RANKL treatment, OC differentiation of RAW 264.7 cells produced large, round, and red-stained multinucleated OCs in the PBS group (Figure 3D). In contrast, after curcumin treatment, the number of mature TRAP-positive OCs decreased (Figure 2E, *p* < 0.05), signifying that curcumin could attenuate OC formation, which was consistent with our previously study (An et al., 2018). Similarly, the number of TRAP-positive OCs significantly decreased after ZSCNP treatment (Figure 3E, *p* < 0.05), indicating that curcumin in ZSCNPs could effectively reduce OC formation, which was in line with the *in vitro* curcumin release experiment in this study.

### *In Vivo* Pharmacokinetics and Distribution

To maximize accumulation of NPs in the target region, it is important to prolong the blood circulation time. Therefore, the blood retention time of the ZSCNPs with an external magnet field (dual-targeting group) was evaluated, and SCNPs (non-targeting group) were used as the control (Supplementary Figure S4). After the 1 h time point, the concentration of SCNPs in blood decreased to 12.4% of the injected dose per gram of tissue (ID/g), and the half-life of SCNPs was 0.5–1 h. After the first 24 h time point, the concentration of SCNPs in blood was reduced to 4.7% ID/g. In contrast, in the dual-targeting group, the concentration of ZSCNPs in blood was still as high as 43.4% ID/g at the 1 h time point and then decreased to 26.4% ID/g at the 6 h time point and to 24.0% ID/g at the 12 h time point, and the half-life of SCNPs was 6–12 h. After the first 24 h time point, the concentration of ZSCNPs in blood was maintained at 22.0% ID/g, and even at the third 24 h time point, the concentration was reduced to 5.3% ID/g, which was still slightly higher than that of SCNPs at the first 24 h time point. Compared with the non-targeting group, the concentration of ZSCNPs with MF targeting was significantly higher than that of SCNPs at each tested time point. These results clearly demonstrate that ZSCNPs with zoledronate anchoring and MF targeting effectively prolonged the blood circulation time in comparison to non-targeting SCNPs, which may be attributed to the high bone targeting affinity of the zoledronate-anchored PLGA-ZOL NPs (Ramanlal Chaudhari et al., 2012) and the passive magnetic targeting strategy of accelerating aggregation of these magnetic response NPs in the target region (Wang et al., 2018).

**FIGURE 4:**
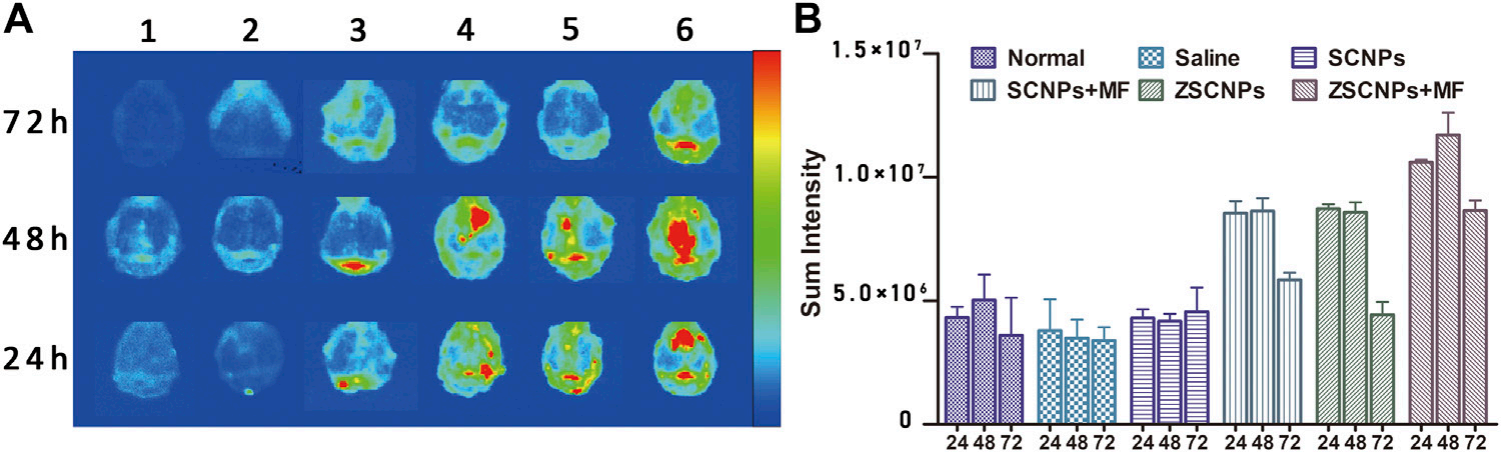
*Ex vivo* NIR fluorescence images of calvarial bones and major organs. **(A)** The *ex vivo* NIR fluorescence images and **(B)** averaged NIR fluorescence intensities of calvarial bones at 24, 48 and 72 h after different treatments: 1) Normal, 2) Saline, 3) SCNPs, 4) SCNPs + MF, 5) ZSCNPs, 6) ZSCNPs + MF.

To evaluate the time point resulting in the best distribution of ZSCNPs on calvarial bones, an *ex vivo* NIRF imaging study was carried out. Nine healthy BALB/c mice and forty-five calvarial osteolysis mice were used for *ex vivo* fluorescence imaging at 24, 48 and 72 h. At different time points, the calvarial bones were harvested and imaged. As shown in Figures 4A,B, the basic fluorescence signals of calvarial bones displayed a very weak blue light, with intensities less than 5.0 × 10^6^ at all the time points in group (1) and group (2). In group (3) treated with non-targeting SCNPs, only a few fluorescence signals were present on calvarial bones. Nevertheless, the magnetic targeting group (4) and zoledronate-anchoring group (5) showed distinctly stronger fluorescence signals on calvarial bones than the non-targeting group (3), indicating that not only zoledronate anchoring but also the MF targeting strategy contributed to the aggregation of NPs in the calvarial bones. Furthermore, the fluorescence signals of the calvarial bones in group (6) treated with ZSCNPs and MF targeting were obviously stronger than those in the other treated groups at each time point, especially at the 24 and 48 h time points, with intensities of more than 1.0 × 10^7^. Compared with the 24 and 72 h time points, the fluorescence signal on calvarial bones at the 48 h time point demonstrated the best signal intensity, which provided an optimal therapeutic time for the *in vivo* therapy study.

### Zoledronate-Anchored, Dual-Targeted, Drug-Loaded Nanoparticles Suppresses Polyethylene-Induced Osteolysis *In Vivo*

**FIGURE 5:**
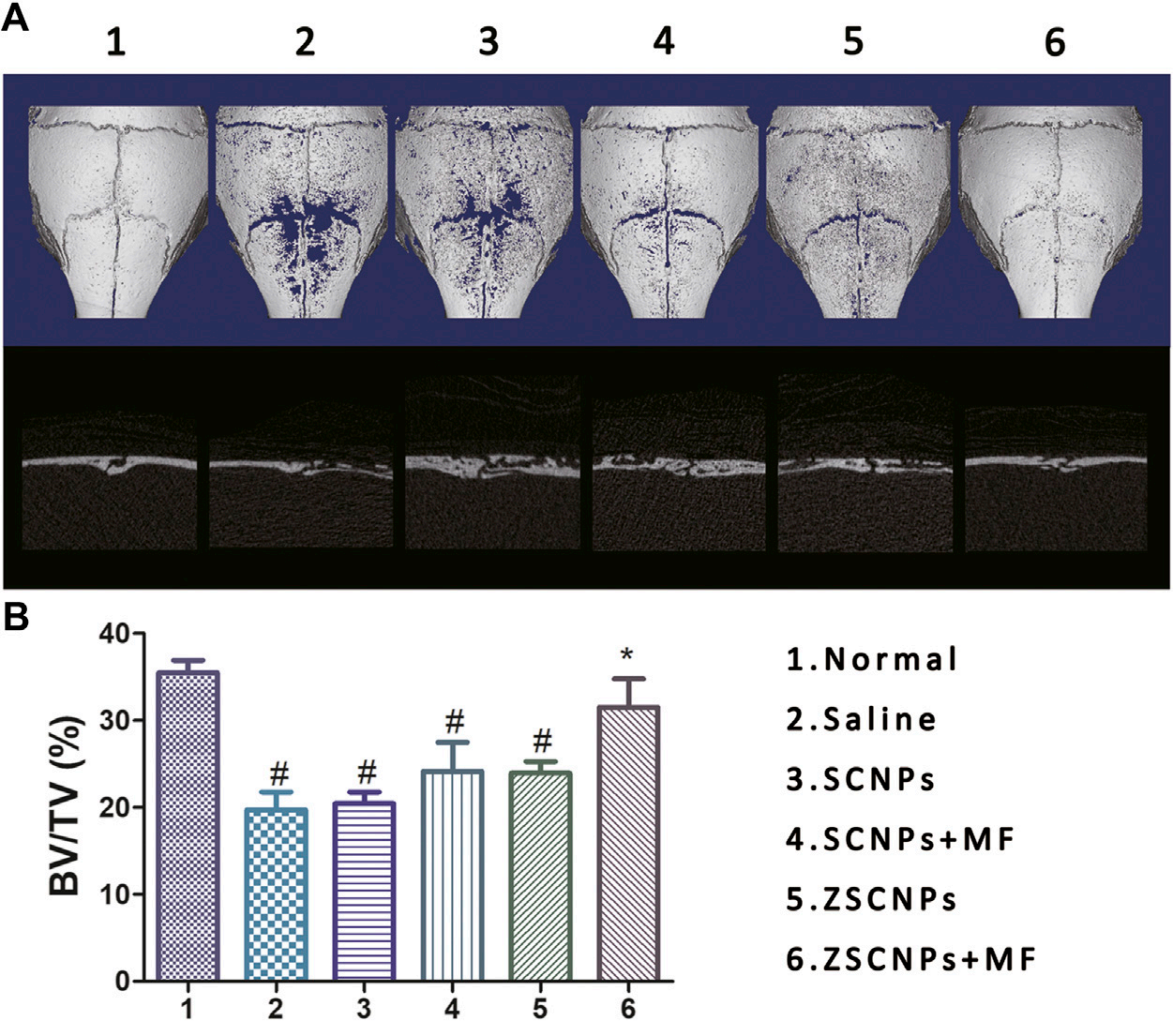
Micro CT images of calvarial bones. **(A)** The *ex vivo* micro CT images and **(B)** corresponding bone volume/tissue volume indices of calvarial bones at 48 h after different treatments: 1) Normal, 2) Saline, 3) SCNPs, 4) SCNPs + MF, 5) ZSCNPs, 6) ZSCNPs + MF. #*p* < 0.05 compared with normal group, *p < 0.05 compared with saline group.

To investigate the *in vivo* effects of ZSCNPs on the pathological formation of OCs during PE-induced osteolysis, calvarial osteolysis mice and air-porch bone graft mice were used. First, bone destruction of calvarial bones was visualized via micro CT imaging. As shown in Figures 5A,B, groups (2) and (3) showed obvious bone erosion, and the bone loss index (BV/TV) in the two groups was not significantly different (*p* > 0.05), indicating that the non-targeting SCNPs have no obvious effect on attenuating PE-induced osteolysis. By contrast, the extent of bone erosion was notably decreased in the magnetic targeting group (4) and zoledronate-anchoring group (5) compared to the non-targeting group (3) (Figure 4A), revealing that both the zoledronate anchoring and MF targeting strategy contributes to an improvement in the therapeutic effect for suppressing PE-induced osteolysis, which supports our *ex vivo* NIR fluorescence imaging results. Although the BV/TV exhibited no significant difference among the three groups (*p* > 0.05) (Figures 5B), the active and passive targeting methods have potential for increasing the aggregation of NPs in calvarial bones, which will further attenuate bone loss in PE-induced calvarial osteolysis. Moreover, the extent of bone erosion was further significantly decreased in the dual-targeting group (Anand et al., 2007) compared with that in the other calvarial osteolysis mouse groups (Figures 5A). Compared with the non-targeting group (3), the BV/TV in group (6) was significantly higher (*p* < 0.05) (Figures 5B), demonstrating that the combination of the active and passive targeting methods has the optimal therapeutic effect for inhibiting PE-induced osteolysis.

**FIGURE 6:**
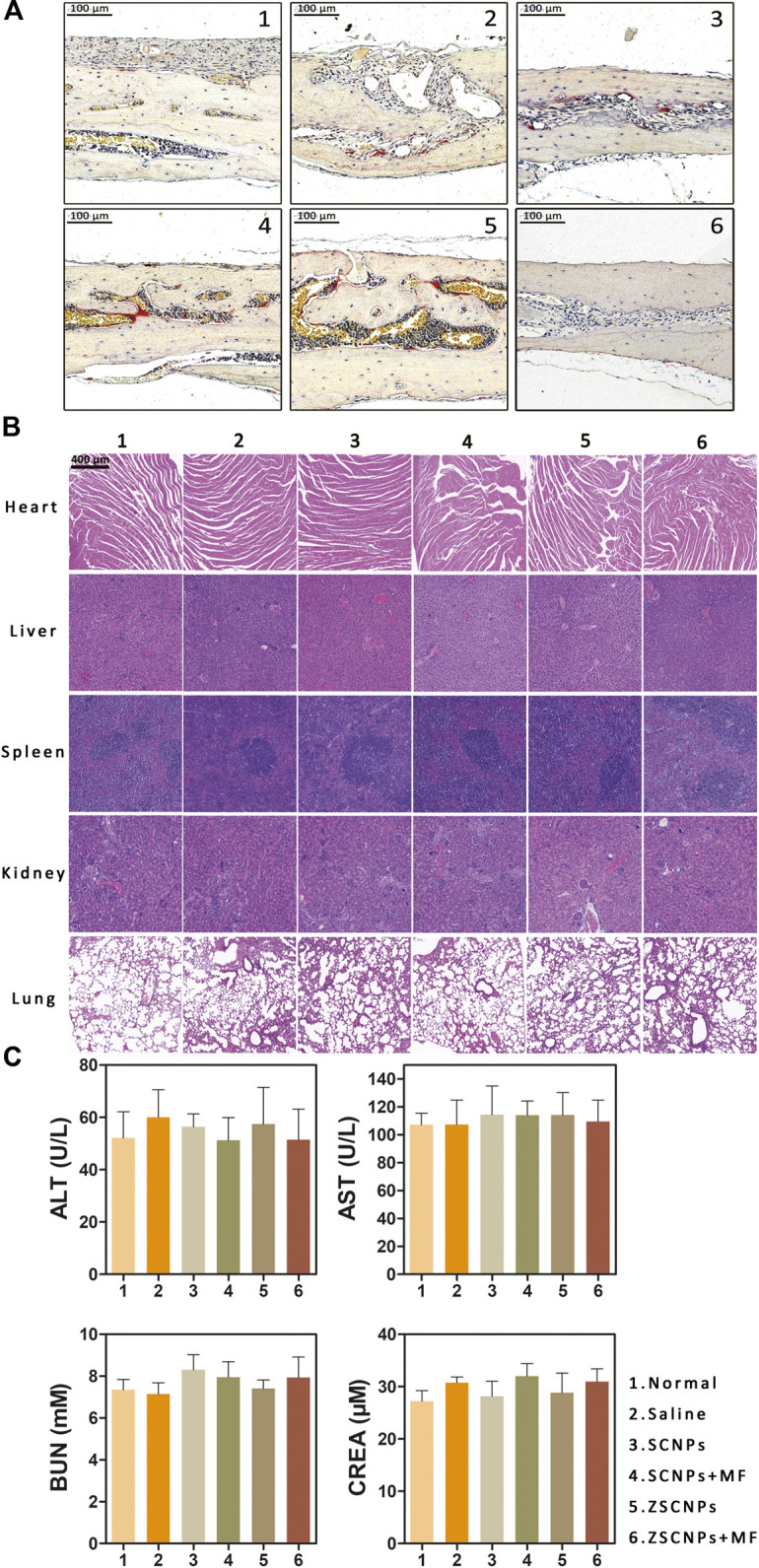
ZSCNPs suppresses polyethylene (PE)-induced calvarial osteolysis. **(A)** tartrate-resistant acid phosphat staining of calvarial bones in PE-induced osteolysis mice after different treatments: 1) Normal, 2) Saline, 3) SCNPs, 4) SCNPs + MF, 5) ZSCNPs, 6) ZSCNPs + MF (scale bar = 100 µm). **(B)** The HE sections of heart, liver, spleen, kidney, and lung harvested from mice after different treatment (scale bar = 50 µm). **(C)** Hepatic functional markers (ALT and AST) and renal functional markers (BUN and CREA) in the blood at 14 days after different treatments.

Histological evaluation of TRAP staining further supported the micro CT findings showing the region of PE-induced calvarial osteolysis after different treatments. TRAP staining revealed mature red-stained multinucleated OCs around eroded bone lesions in groups (2–5) (Figures 6A). ZSCNPs with MF targeting treatment in group (6) significantly reduced the area of osteolytic lesions, with a diminished number of TRAP-positive OCs in calvaria osteolysis (Figures 6A), indicating that the dual-targeting ZSCNPs could enhance NP distribution in bone loss regions, increase the arrival concentration of the released curcumin in NPs and reverse osteoclastogenesis of OCs, thus showing strong potential for future clinical translation for suppressing PE-induced osteolysis. In addition, H&E staining analysis of major organs (heart, liver, spleen, kidney, and lung) revealed no obvious damage (Figures 6B), and the levels of hepatic and renal function markers (ALT, AST, BUN and CREA) in the blood also showed no significant changes after the different treatments (Figures 6C) (*p* > 0.05), indicating that ZSCNPs were safe and non-toxic to normal tissues.

**FIGURE 7:**
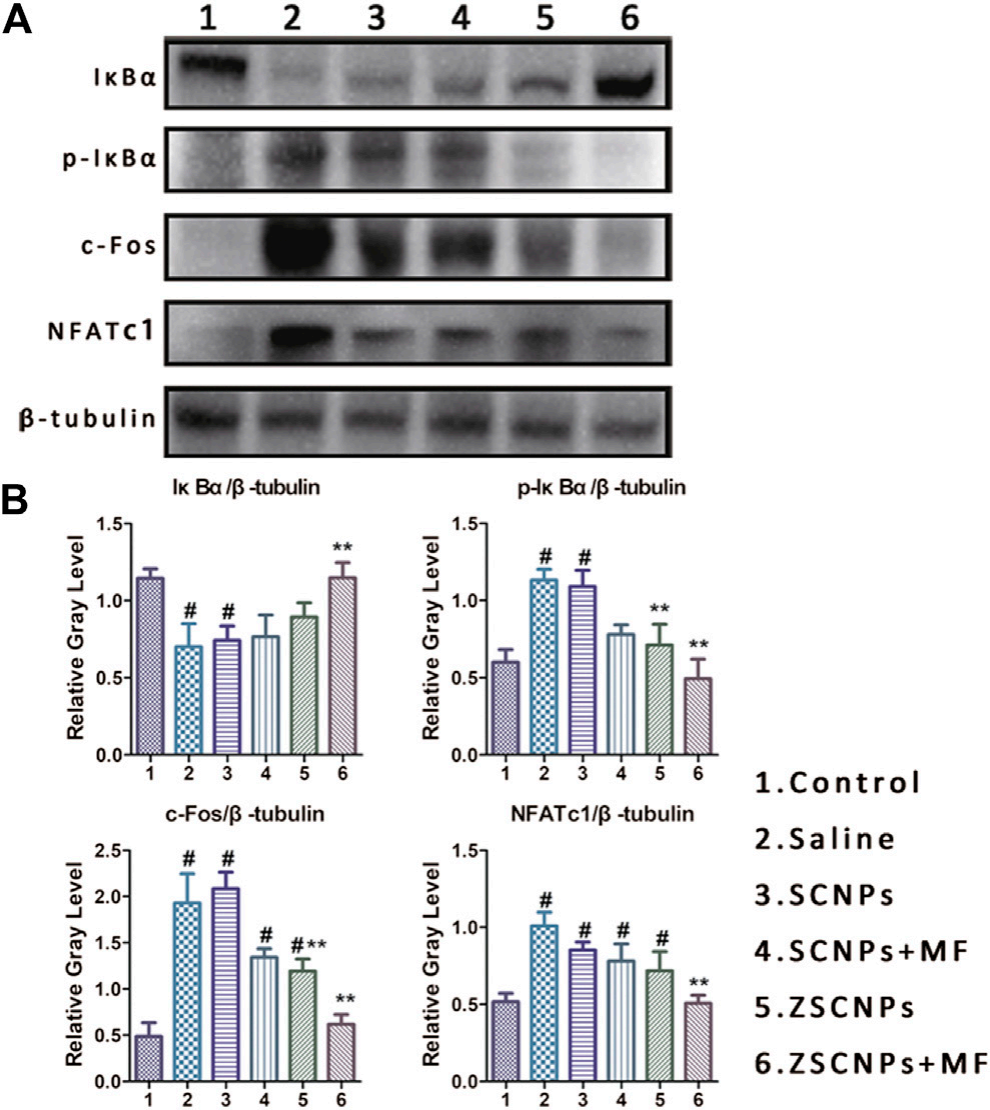
ZSCNPs suppresses polyethylene-induced osteolysis via repressing NF-κB signaling. **(A)** The expressions of IκBα, *p*-IκBα, c-Fos and uclear factor of activated T cells cytoplasmic 1 in air-porch wall tissues using western blot analysis after different treatments: 1) Control, 2) Saline, 3) SCNPs, 4) SCNPs + MF, 5) ZSCNPs, 6) ZSCNPs + MF. **(B)** Relative protein expression was normalized to the corresponding β-tubulin. #*p* < 0.05 compared with control group, **p < 0.05 compared with saline group.

To further investigate the mechanism by which ZSCNPs suppress PE-induced osteolysis, air-porch bone graft mice were used. Whole air-porch samples were collected and the proteins of air-porch wall tissues were extracted for further assays on the 14th day. As an important player in NF-κB activation, IKK can induce IκBα phosphorylation and degradation and activate the RANK/c-Fos/NFATc1 signaling pathway. However, some studies have reported that curcumin can inhibit the phosphorylation and degradation of IκBα by inhibiting the effect of IKK and finally achieve inhibition of the entire NF-κB activation process (Bharti et al., 2004; An et al., 2018). The expression of IκBα, *p*-IκBα, c-Fos and NFATc1 in air-porch wall tissues was assessed using western blot analysis after different treatments (Figure 7A). The results of statistical analysis of the relative gray levels are shown in Figure 7B. As shown in Figures 7A,B, IκBα protein showed the lowest expression and *p*-IκBα protein displayed the highest expression, indicating activation of the NF-κB process with the phosphorylation and degradation of IκBα in the saline group (2). Subsequently, the expression of c-Fos and NFATc1 exhibited the highest expression in group (2) due to activation of the RANK/c-Fos/NFATc1 signaling pathway. In contrast, the decrease in IκBα protein expression levels was attenuated by curcumin in groups (3-6), especially in group (6), and the expression of IκBα protein was significantly different from that in group (2) (*p* < 0.05). Similarly, the increase in the *p*-IκBα protein expression level was attenuated by curcumin in groups (3‒6), and the expression of *p*-IκBα protein in group (6) was significantly lower than that in group (2) (*p* < 0.05), demonstrating that curcumin effectively inhibited the effect of IKK. Moreover, the increase in the c-Fos and NFATc1 protein expression levels was attenuated by curcumin in groups (3‒6) to different extents, and the expression of c-Fos and NFATc1 proteins in group (6) was markedly lower than that in group (2) (*p* < 0.05), indicating that curcumin efficiently repressed the entire NF-κB activation process. These results demonstrate that the dual-targeting ZSCNPs successfully suppressed PE-induced osteolysis via the RANK/c-Fos/NFATc1 signaling pathway to the greatest extent, which was in strong agreement with the micro CT imaging and TRAP staining findings in this study.

## Conclusion

Novel dual-targeted, curcumin-loaded PLGA NPs (ZSCNPs) with excellent biocompatibility and wonderful bone binding affinity were developed to inhibit PE-induced osteolysis. Under external magnetic field guidance, the ZSCNPs accurately targeted to osteolytic sites, showed sustained curcumin release, efficiently suppressed the effect of IKK, effectively inhibited the NF-κB signaling pathway activation process, and ultimately prevented OC formation and PE-induced osteolysis. Therefore, these novel dual-targeted, drug-loaded NPs will undoubtedly extend clinically treatment of PPO after TJA.

## Data Availability Statement

The original contributions presented in the study are included in the article/Supplementary Material, further inquiries can be directed to the corresponding author.

## Ethics Statement

The animal study was reviewed and approved by Ethics Committee of Central South University.

## Author Contributions

LW and CN: conceptualization and data curation. JL and CN: methodology. JL, ZJ, ZZ, YP, QX, QG, SA, and YH: analysis and investigation. CN: writing original draft preparation. LW: writing-review and editing. YH and LW: supervision.

## Funding

This project was funded by the National Natural Science Foundation of China (Grant No. 81601883 and 81974267), Hunan Provincial Natural Science Foundation of China (Grant 2018JJ3861) and Hunan Provincial Health Commission Research Foundation Project (B2019166) and the Fundamental Research Funds for the Central Universities of Central South University.

## Conflict of Interest

The authors declare that the research was conducted in the absence of any commercial or financial relationships that could be construed as a potential conflict of interest.
