# Supplementary material for: Targeted Delivery of Curcumin to Polyethylene-Induced Osteolysis by Magnetically Guided Zoledronate-Anchored Poly Lactic-Co-Glycolic Acid Nanoparticles via Repressing NF-κB Signaling
Source: Front Pharmacol. 2020 Dec 4;11:600156. doi: 10.3389/fphar.2020.600156 (PMC7747869; doi:10.3389/fphar.2020.600156)
Supplement: Supplementary file 1 [file datasheet1.docx]

**Supporting Information**

**Targeted delivery of curcumin to polyethylene-induced osteolysis by magnetically guided zoledronate-anchored PLGA nanoparticles via repressing NF-κB signaling**

Jingyi Li ^a,b,1^, Chengcheng Niu ^c,d,1^, Zichao Jiang ^a,b^, Zhen Zhang ^a,b^, Yixiao Pan ^a,b^, Qiqi Xing ^a,b^, Qi Guo ^a,b^, Senbo An ^a,b^, Yihe Hu ^a,b^, Long Wang ^a,b,^*

**^a^** Department of Orthopedics, Xiangya Hospital, Central South University, Changsha, Hunan 410008, China

**^b^** Hunan Engineering Research Center of Biomedical Metal and Ceramic Implants, Department of Orthopedics, Xiangya Hospital, Central South University, Changsha, Hunan 410008, China

**^c^** Department of Ultrasound Diagnosis, Second Xiangya Hospital, Central South University, Changsha, Hunan 410011, China

**^d^** Research Center of Ultrasonography, Second Xiangya Hospital, Central South University, Changsha, Hunan 410011, China

**Address all correspondence to:** Long Wang, Department of Orthopedics, Xiangya Hospital, Central South University, Changsha, Hunan, China, 410008; E-mail: [dr_wanglong@csu.edu.cn](mailto:dr_wanglong@csu.edu.cn)

^1^ These authors contributed equally to this work.


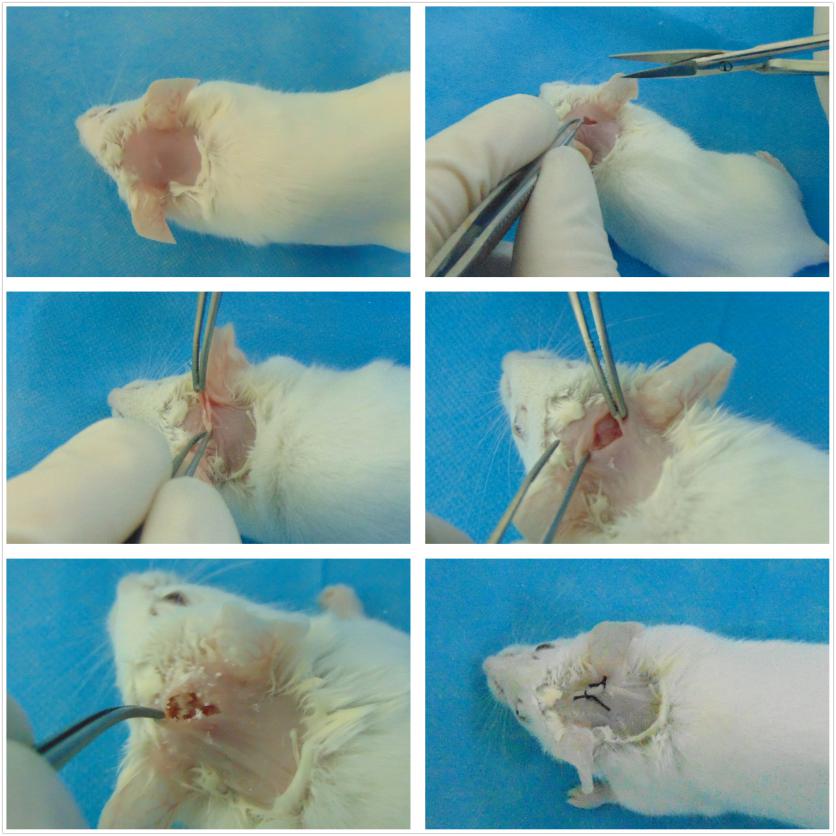


Fig. S1 Photos of the mice calvarial osteolysis model establishment.


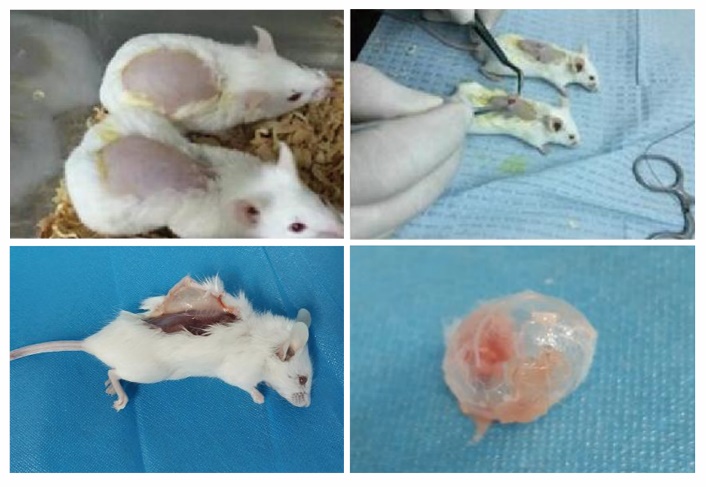


Fig. S2 Photos of the mice air-porch bone graft model establishment.
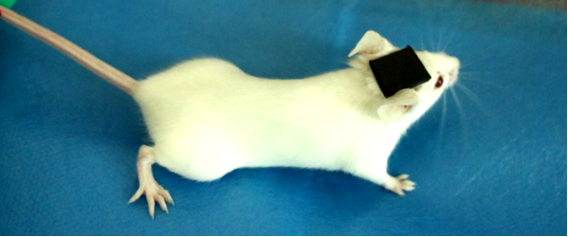


Fig. S3 Photograph of in vivo magnet targeting experiment.


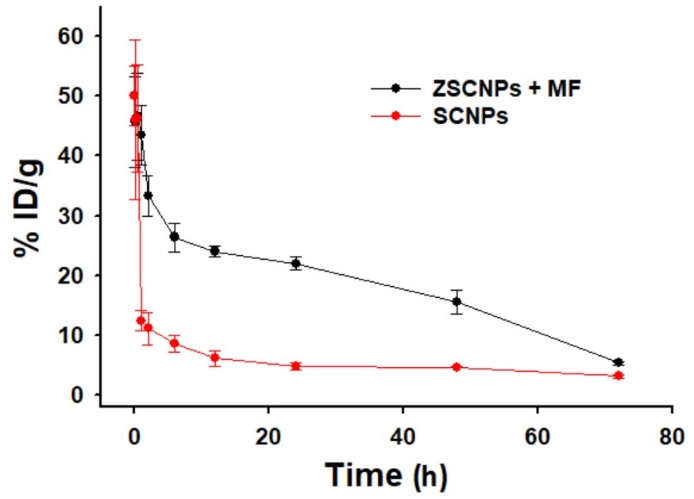


Fig. S4 In vivo pharmacokinetic curves over a span of 72 hours after intravenous injection of SCNPs (non-targeting group) or ZSCNPs with a magnet field targeting (dual targeting group).
